# A novel function of hepatocyte growth factor in the activation of checkpoint kinase 1 phosphorylation in colon cancer cells

**DOI:** 10.1007/s11010-017-3075-0

**Published:** 2017-06-01

**Authors:** Na Song, Xiaofang Che, Lu Xu, Jinglei Qu, Huachuan Zheng, Kezuo Hou, Xiujuan Qu, Yunpeng Liu

**Affiliations:** 1 Department of Medical Oncology, The First Hospital of China Medical University, Shenyang, 110001 People’s Republic of China; 2 Key Laboratory of Anticancer Drugs and Biotherapy of Liaoning Province, The First Hospital of China Medical University, Shenyang, 110001 People’s Republic of China; 3 Department of Experimental Oncology, Shengjing Hospital of China Medical University, Shenyang, Liaoning 110001 People’s Republic of China

**Keywords:** HGF, MET, Chk1, AKT, Colon cancer

## Abstract

The ATR/checkpoint kinase 1 (Chk1) pathway plays an essential role in modulating the DNA damage response and homologous recombination. Particularly, Chk1 phosphorylation is related to cancer prognosis and therapeutic resistance. Some receptor tyrosine kinases participate in the regulation of Chk1 phosphorylation; however, the effect of hepatocyte growth factor (HGF) on Chk1 phosphorylation is unknown. In the present study, we demonstrated that HGF moderately activated Chk1 phosphorylation in colon cancer cells by upregulating TopBP1 and RAD51, and promoting TopBP1–ATR complex formation. Furthermore, AKT activity, which was promoted by HGF, served as an important mediator linking HGF/MET signaling and Chk1 phosphorylation. Depleting AKT activity attenuated basal expression of p-Chk1 and HGF-induced Chk1 activation. Moreover, AKT activity directly regulated TopBP1 and RAD51 expression. AKT inhibition suppressed HGF-induced upregulation of TopBP1 and RAD51, and enhanced TopBP1/ATR complex formation. Our results show that HGF was involved in regulating Chk1 phosphorylation, and further demonstrate that AKT activity was responsible for this HGF-induced Chk1 phosphorylation. These findings might potentially result in management of prognosis and therapeutic sensitivity in cancer therapy.

## Introduction

DNA damage induced by radiation and chemotherapy drugs promotes cell cycle arrest via activation of cell cycle checkpoints, allowing time for DNA repair to maintain genomic integrity [1, 2]. In most cancer cells, the G1 checkpoint is defective because of p53 mutations; therefore, the G2 checkpoint is impaired and necessary for the cell to respond to DNA damage. The two most important features of G2 checkpoint activation are ataxia-telangiectasia mutated (ATM) and ATM- and rad3-related (ATR) signaling, which activate their respective downstream substrates checkpoint kinase 2 (Chk2) and checkpoint kinase 1 (Chk1) [3]. Chk1 is the direct substrate of ATR and is rapidly phosphorylated on serine 317 and serine 345 by ATR under replication stress [2]. In contrast with ATM, which is only activated by DNA double-strand breaks, ATR is exclusively activated by breaks in single-stranded DNA [4].

Recent studies have revealed that Chk1 phosphorylation has unique clinical implications, including for cancer prognosis and therapeutic sensitivity. In metastatic brain tumors, there was a strong inverse relationship between phosphorylated Chk1 (p-Chk1) level and progression-free survival [5]. Furthermore, an evaluation of p-Chk1 protein expression in 1712 breast cancer cases revealed that high cytoplasmic p-Chk1 levels were significantly associated with poor breast cancer-specific survival and adverse outcome [6]. Thus, the relationship between prognosis and p-Chk1 level in other types of cancer is worthy of further investigation. In addition to the emerging prognostic role for expression of p-Chk1, previous studies have demonstrated that elevated p-Chk1 expression reduces sensitivity to radiotherapy and chemotherapy [7–9]. Inhibition of ATR/Chk1 has cytotoxic effects and renders cancer cells, especially p53-deficient cancer cells, more sensitive to radiation and to a variety of chemotherapy agents [9–11]. However, the mechanisms by which Chk1 phosphorylation is modulated by common pathways involved in tumorigenesis and cancer progression have not been fully elucidated.

Transmembrane receptor tyrosine kinases (RTKs) play important roles in cell proliferation and survival. Recent studies indicate that some RTKs are involved in regulation of the DNA damage response. HER RTKs, the most common RTK family, were activated on exposure to irradiation [12]. Among the four HER RTK family members, inhibition of HER2 abolished irradiation-induced activation of ATM/ATR signaling and ERK phosphorylation, which suggests a vital upstream role of HER2 in regulating the G2 checkpoint response [12]. However, the detailed mechanism of the effect of HER2 on ATR/Chk1 signaling is not well defined. Contrary to the above study, an earlier report proposed that EGF negatively regulated Chk1 phosphorylation via regulation of Mig-6 [13]. Recently, activity of another common RTK, insulin-like growth factor 1 receptor (IGF-1R), was found to enhance repair of ultraviolet B-induced DNA damage in human keratinocytes [14]. In our previous study, we demonstrated a role of the hepatocyte growth factor (HGF)/MET pathway in the regulation of colon cancer cell proliferation and cetuximab resistance [15]. Furthermore, emerging data from recent studies have demonstrated that deregulated MET activity led to radiosensitization via downregulation of the ATR/Chk1 pathway, especially in p53-deficient cancer cells [16–18]. However, the possible involvement of HGF in the regulation of Chk1 activation remains to be elucidated.

Although some studies have demonstrated that RTKs participate in regulation of the ATR/Chk1 pathway and repair of DNA damage, the factors that directly mediate this regulation have not been confirmed. Previous studies suggested that AKT and ERK, which are essential downstream molecules of RTKs, modulated Chk1 function and the response to DNA damage [12, 19]. Notably, AKT participated in regulation of both cell proliferation and genome stability, modifying both the response to and repair of genotoxic damage in complex ways [20]. However, the suggestion that AKT modulates Chk1 activity is controversial. Previous studies indicated that AKT suppressed DNA damage processing and inhibited Chk1 activity by blocking the physical interaction between Chk1 and Claspin, or by preventing association of ATR with TopBP1 by inducing TopBP1 oligomerization [19, 21, 22]. However, another study showed that inhibition of the PI3K/AKT/GSK3β pathway downregulated DNA damage-induced Chk1 activation [23]. Moreover, AKT kinase inactivation decreased RAD51 expression, which was implicated in the repair of homologous recombination [24]. Therefore, the effect of AKT on Chk1 activity requires further investigation.

In this study, we explored the role of HGF on the ATR/Chk1 pathway in colon cancer cells. We demonstrated that AKT is a key mediator of HGF-induced Chk1 phosphorylation, and that TopBP1 and RAD51 were also involved in regulating this process. These results provide new insight into the interaction of the HGF/MET pathway with Chk1 activity and reveal the complexity of Chk1 activity in cancer progression and treatment.

## Materials and methods

### Cell culture and reagents

Colon cancer cells HT-29 and HCT-116 were obtained from the Type Culture Collection of the Chinese Academy of Sciences (Shanghai, China). Both cells were cultured in Roswell Park Memorial Institute (RPMI) 1640 medium with 10% fetal bovine serum. HGF was purchased from R&D systems. LY294002 and hydroxyurea (HU) were purchased from Sigma-Aldrich. Antibodies to MET, phospho-MET (Tyr1234/1235), phospho-Chk1 (S345), AKT, phospho-AKT (Ser473), RAD51, poly (ADP-ribose) polymerase (PARP) were obtained from Cell Signaling Technology. Antibodies to Chk1, ATR, TopBP1, and Claspin were obtained from Bethyl. Antibodies to β-Tubulin and RAD51 (ab88572) were purchased from Abcam. Anti-Actin, secondary goat anti-rabbit, and goat anti-mouse antibodies were purchased from Santa Cruz Biotechnology.

### Western blotting

Western blotting was performed as described in our previous studies [15].

### Small interfering RNA transfections

The siRNA sequences were from View solid biotechnology co., LTD (Beijing, China). The MET siRNA sequence was 5′-GCCUGAAUGAUGACAUUCU-3′. TopBP1 siRNA sequences were 5′-GCUCUGUAAUAGUCGACUAtt-3′ and 5′-GGAUAUAUCUUUGCGGUUUtt-3′. RAD51 siRNA sequences were 5′-GGUAGAAUCUAGGUAUGCAtt-3′ and 5′-CCAGCUCCUUUAUCAAGCAtt-3′. AKT siRNA sequence was 5′-CUCACAGCCCUGAAGUACUtt-3′. The siRNAs were transfected with Lipofectamine 2000 (Invitrogen, Carlsbad, CA, USA) per the manufacturer’s instructions.

### Subcellular fractionation

The cytosolic and nuclear proteins were carried out using the Nuclear Extract kit (Active Motif) according to the manufacturer’s instructions. The fractions were quantified using the BCA assay, then detected by Western blotting.

### Co-immunoprecipitation

Co-immunoprecipitations were performed using 200 μg of cell lysates with 4 μl of anti-MET, anti-ATR antibody, control IgG Mouse, or Rabbit mixed with Protein A Agarose beads (GE Healthcare Bio-Sciences AB, Pittsburgh, USA).The final mixture was gently rocked overnight at 4 °C. Then the beads were spun down for 1 min at 13,000/rpm and washed four times with lysis buffer. Finally, 40 μl 2× sampling buffer was added and boiled at 95 °C for 5 min.

### Fluorescence microscopy

HT-29 cells were seeded at 100,000 cells/well and treated with 25 ng/ml HGF for 8 h in Lab-Tek chamber slides (Nunc S/A, Polylabo, Strasbourg, France). Then the cells were fixed with 3.3% para-formaldehyde for 20 min, permeabilized with PBS buffer containing 0.2% Triton X-100 for 5 min and blocked with 5% bovine serum albumin (BSA) for 30 min. The slides were incubated with anti-RAD51 antibody overnight at 4 °C and then incubated with fluorescein isothiocyanate (FITC)-conjugated goat anti-rabbit IgG for 1 h. The nucleus was counter-stained with DAPI for 5 min, then observed under a fluorescence microscope (BX53; Olympus Corporation, Tokyo, Japan).

### Reverse-transcription-polymerase chain reaction (RT-PCR)

Total RNA was isolated with the RNeasy mini kit (Qiagen, Carlsbad, CA, USA). RT-PCR was performed with primer pairs for TopBP1: forward (5′-AAGAGTTTCCTTGTTTTGGG-3′) and reverse (5′-CATGCCTTTCTTTGCATTGG-3′); primer pairs for RAD51: forward (5′-CAACCCATTTCACGGTTAGAGC-3′) and reverse (5′-GCTTTGGCTTCACTAATTCCCT-3′). For GAPDH as control: forward (5′-GGTGAAGGTCGGAGTCAACGG-3′) and reverse (5′-GAGGTCAATGAAGGGGTCATTG-3′). PCR conditions were 95 °C for 5 min; 35 cycles of 95 °C for 30 s, 58 °C for 34 s, 72 °C for 30 s; one cycle of 72 °C for 10 min. Then the amplified products were separated on 1.5% agarose gels, and stained with ethidium bromide and visualized under UV illumination.

## Results

### HGF induced Chk1 phosphorylation in colon cancer cells

**Fig. 1:**
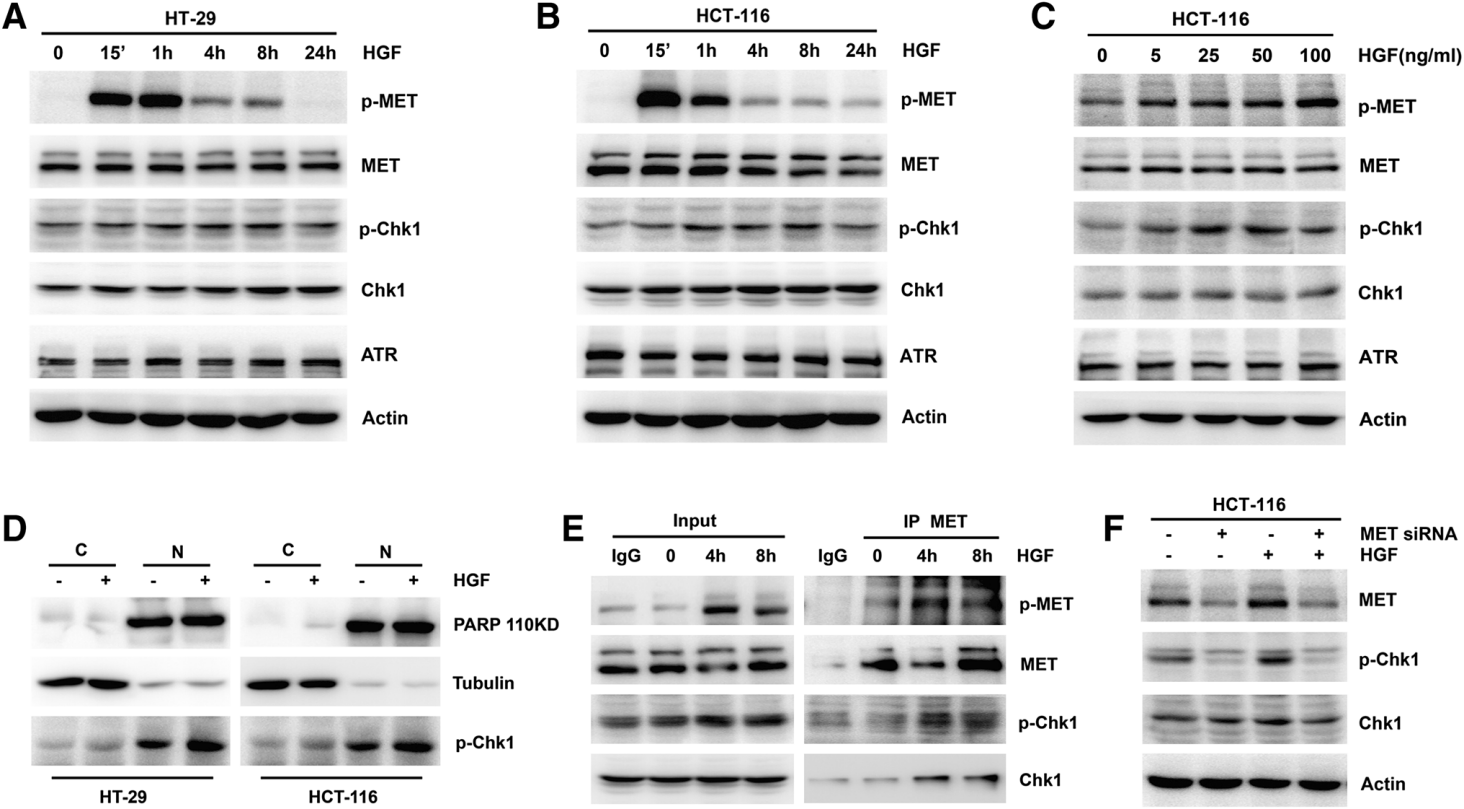
Effects of HGF on Chk1 phosphorylation in colon cancer cells. **a**, **b** HGF (25 ng/ml) was added to HT-29 and HCT-116 cells for the indicated times. The expression of MET, MET phosphorylation, Chk1, Chk1 phosphorylation, and ATR was performed by Western blotting. For all Western blotting, actin was used as a loading control. **c** Indicated concentrations of HGF (5, 25, 50 and 100 ng/ml) was added to HCT-116 cells for 4 h. The expression of MET, MET phosphorylation, Chk1, Chk1 phosphorylation and ATR was performed by Western Blotting. **d** HT-29 and HCT-116 cells were treated with 25 ng/ml HGF for 8 h, then subjected to subcellular fractionation. The expression of PARP, Tubulin and Chk1 phosphorylation in the cytosol (C) and nuclear (N) fractions was analyzed by Western blotting. **e** HCT-116 cells were treated with 25 ng/ml HGF for 4 and 8 h. Whole-cell extracts from HCT-116 cells were immunoprecipitated with anti-MET antibody. The immunoprecipitates were probed with total and phosphorylation of MET and Chk1 antibodies. Input represents cell lysates that were not subjected to immunoprecipitation. IgG mouse was used as control. **f** HCT-116 cells were transiently transfected with scramble control siRNA or MET siRNA, then added with 25 ng/ml HGF for 4 h. The expression of total MET, Chk1 and Chk1 phosphorylation was performed by Western blotting

To establish the effect of HGF on Chk1 phosphorylation, we selected two representative colon cancer cell lines: HT-29 (wild-type RAS, p53-deficient) and HCT-116 (mutant RAS, wild-type p53, ATM deficient), which are dependent on the ATR/Chk1 pathway. When the cells were treated with HGF for the indicated times at different doses, activation of MET phosphorylation was observed in both cell lines, accompanied by a gradual increase in phosphorylation of Chk1 on Ser345, which promoted Chk1 activation (Fig. 1a–c). Furthermore, to determine the subcellular location of the elevated p-Chk1, we separated the nucleus and cytoplasm. Phosphorylated Chk1 was mainly present in the nucleus, and was activated in both the nucleus and cytoplasm in the presence of HGF (Fig. 1d). Next, to determine whether MET–Chk1 complex formation occurred, we performed a co-immunoprecipitation assay. The results showed that there was no physical interaction between MET and Chk1 without HGF stimulation. After cells were treated with HGF for 4 and 8 h, p-MET and p-Chk1 levels were elevated. Moreover, a MET–Chk1 complex began to form after 4 h of HGF induction (Fig. 1e). To further explore the role of the HGF/MET pathway on Chk1 phosphorylation, we used siRNA to downregulate MET expression. Expression of basal Chk1 phosphorylation and induction of Chk1 activation by HGF were both partially inhibited when MET was downregulated (Fig. 1f). These results indicate that HGF stimulated Chk1 phosphorylation in colon cancer cells.

### HGF promoted TopBP1 expression in colon cancer cells

**Fig. 2:**
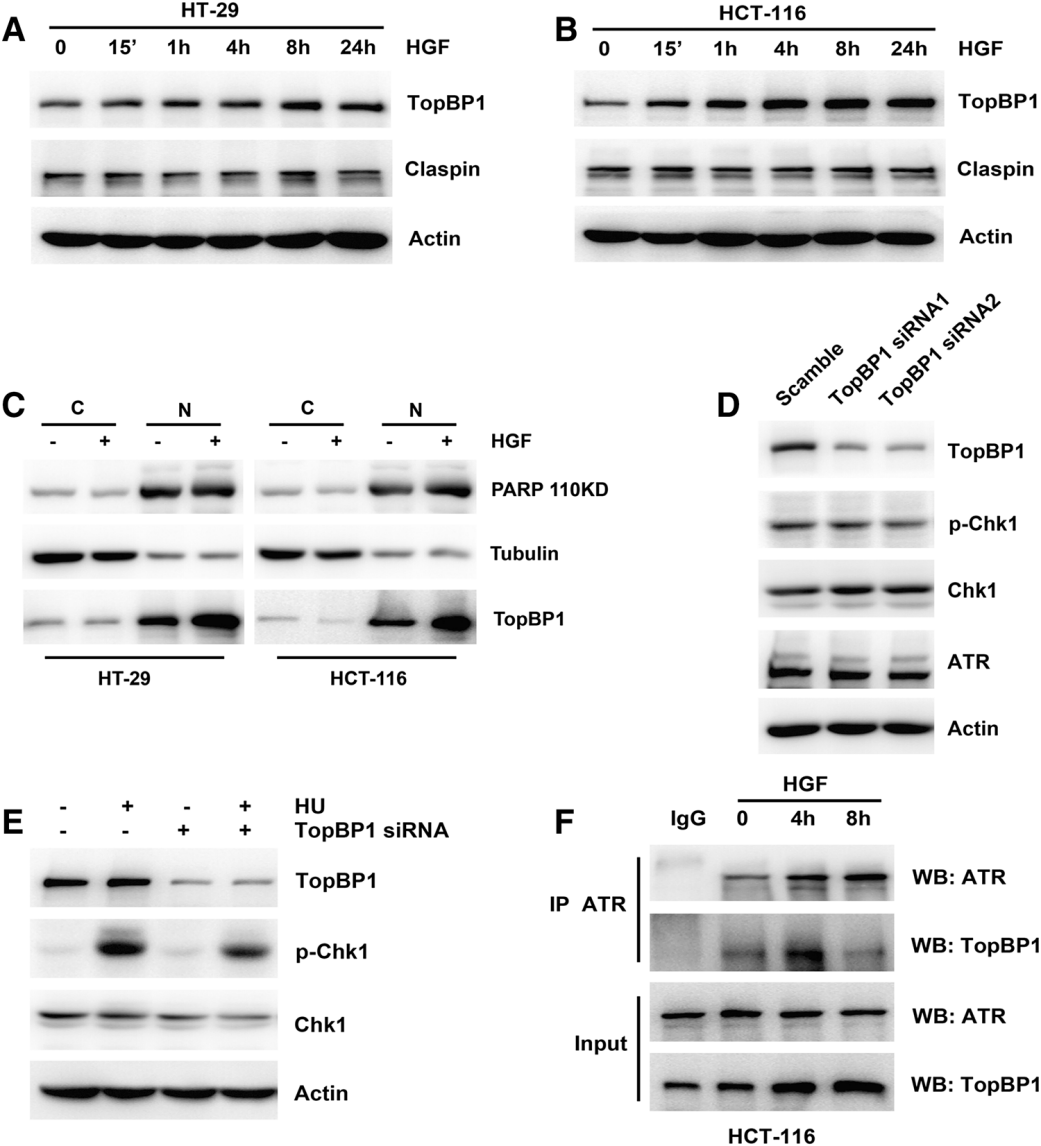
Effects of HGF on expression of TopBP1 in colon cancer cells. **a**, **b** HGF (25 ng/ml) was added to HT-29 and HCT-116 cells for the indicated times. The expression of TopBP1 and Claspin was performed by Western blotting. **c** HT-29 and HCT-116 cells were treated with 25 ng/ml HGF for 8 h, then subjected to subcellular fractionation. The expression of PARP, tubulin and TopBP1 in the cytosol (C) and nuclear (N) fractions was analyzed by Western blotting. **d** HCT-116 cells were transiently transfected with Scramble Control siRNA or two pairs of TopBP1 siRNA separately for 48 h. The expression of TopBP1, ATR, Chk1, and Chk1 phosphorylation was performed by Western blotting. **e** HCT-116 cells were transiently transfected with scramble control siRNA or TopBP1 siRNA, then added with 1 μM HU for 1 h. The expression of TopBP1, Chk1, and Chk1 phosphorylation was performed by Western Blotting. **f** HCT-116 cells were treated with 25 ng/ml HGF for 4 hand 8 h. Whole-cell extracts from HCT-116 cells were immunoprecipitated with anti-ATR antibody. The immunoprecipitates were probed with ATR and TopBP1 antibodies. Input represents cell lysates that were not subjected to immunoprecipitation. Control immunoprecipitation was done using IgG rabbit

**Fig. 3:**
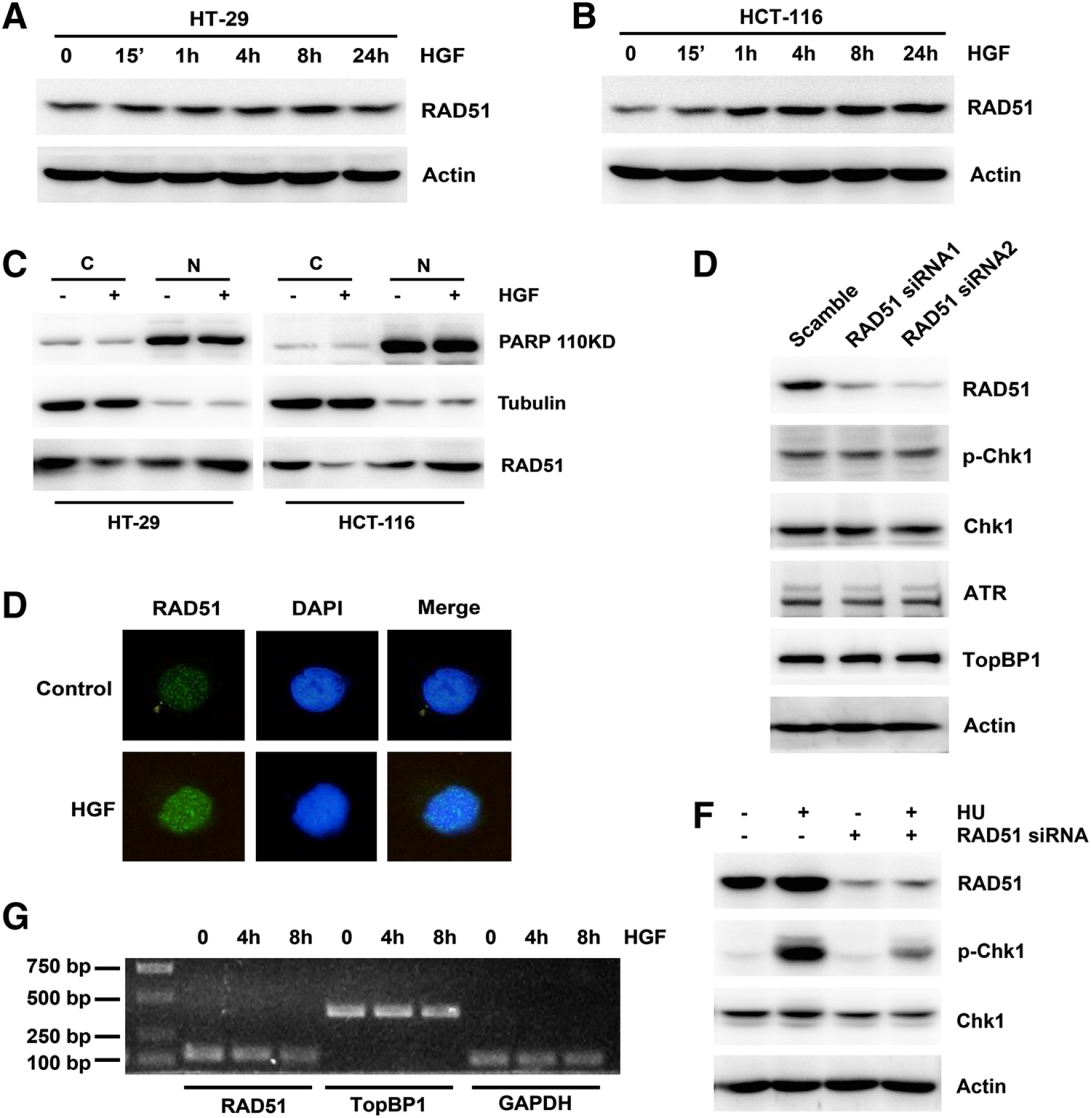
Effects of HGF on expression of RAD51 in colon cancer cells. **a**, **b** HGF (25 ng/ml) was added to HT-29 and HCT-116 cells for the indicated times. The expression of RAD51 was performed by Western blotting. **c** HT-29 and HCT-116 cells were treated with 25 ng/ml HGF for 8 h, then subjected to subcellular fractionation. The expression of PARP, Tubulin and RAD51 in the cytosol (C) and nuclear (N) fractions was analyzed by Western blotting. **d** HT-29 cells were treated with 25 ng/ml HGF for 8 h, and cyto-staining for RAD51 (*green*) and nuclei (*blue*) were detected by immunofluorescence. **e** HCT-116 cells were transiently transfected with scramble control siRNA or two pairs of RAD51 siRNA separately for 48 h. The expression of RAD51, ATR, Chk1, TopBP1, and Chk1 phosphorylation was performed by Western blotting. **f** HCT-116 cells were transiently transfected with scramble control siRNA or RAD51 siRNA, then added with 1 μM HU for 1 h. The expression of RAD51, Chk1 and Chk1 phosphorylation was performed by Western blotting. **g** HCT-116 cells were treated with 25 ng/ml HGF for 4 and 8 h. The mRNA levels of RAD51, TopBP1, and GAPDH were measured by RT-PCR. (Color figure online)

To determine the mechanisms underlying HGF-induced Chk1 phosphorylation, we first examined changes in two important adaptor proteins, TopBP1 and Claspin, which facilitate Chk1 phosphorylation in response to genotoxic stress. When cells were stimulated with HGF, total TopBP1 expression levels gradually increased in both HT-29 and HCT-116 cells, whereas Claspin expression did not obviously change from basal levels (Fig. 2a, b). A subcellular fractionation assay indicated that TopBP1 expression was elevated in the nucleus on HGF exposure (Fig. 2c), and RT-PCR revealed that the level of TopBP1 mRNA remained unchanged (Fig. 3f). To validate the impact of TopBP1 on basal Chk1 expression and Chk1 activation in response to DNA damage, we used siRNA to deplete TopBP1 expression and HU to elevate p-Chk1 levels. TopBP1 downregulation had almost no effect on basal levels of p-Chk1 or ATR (Fig. 2d), but strongly attenuated HU-induced Chk1 phosphorylation (Fig. 2e). Upon DNA damage, TopBP1, ATR, and ATRIP form a complex to facilitate Chk1 activation. We therefore examined the effect of HGF stimulation on TopBP1–ATR complex formation. ATR co-immunoprecipitation revealed a physical interaction between ATR and TopBP1 in HCT-116 cells. The affinity of the two proteins for each other was enhanced after 4 h of HGF stimulation (Fig. 2f). These results reveal that elevated TopBP1 levels and TopBP1–ATR complex formation might contribute to HGF-induced Chk1 activation.

### HGF promoted RAD51 expression in colon cancer cells

Next, to further explore HGF-induced Chk1 activation, we evaluated the expression of RAD51, which regulates Chk1 activity via homologous recombination-dependent DNA repair [25]. We observed that RAD51 expression was remarkably enhanced after HGF stimulation (Fig. 3a, b), and RT-PCR revealed that the RAD51 mRNA levels remained unchanged (Fig. 3g). The HGF-induced increase in RAD51 expression was confirmed by immunofluorescence staining (Fig. 3d), and a subcellular fractionation experiment suggested that RAD51 levels in the cytoplasm apparently declined while RAD51 levels in nucleus increased after addition of HGF (Fig. 3c), thereby indicating that RAD51 translocated from the cytoplasm into the nucleus in response to HGF. Additionally, RAD51 downregulation by siRNA did not change the basal levels of p-Chk1 or ATR (Fig. 3e), but dramatically inhibited HU-induced Chk1 activation (Fig. 3f). These data demonstrate that HGF induced Chk1 activation by enhancing expression of RAD51 and promoting its nuclear translocation.

### AKT regulated Chk1 phosphorylation

**Fig. 4:**
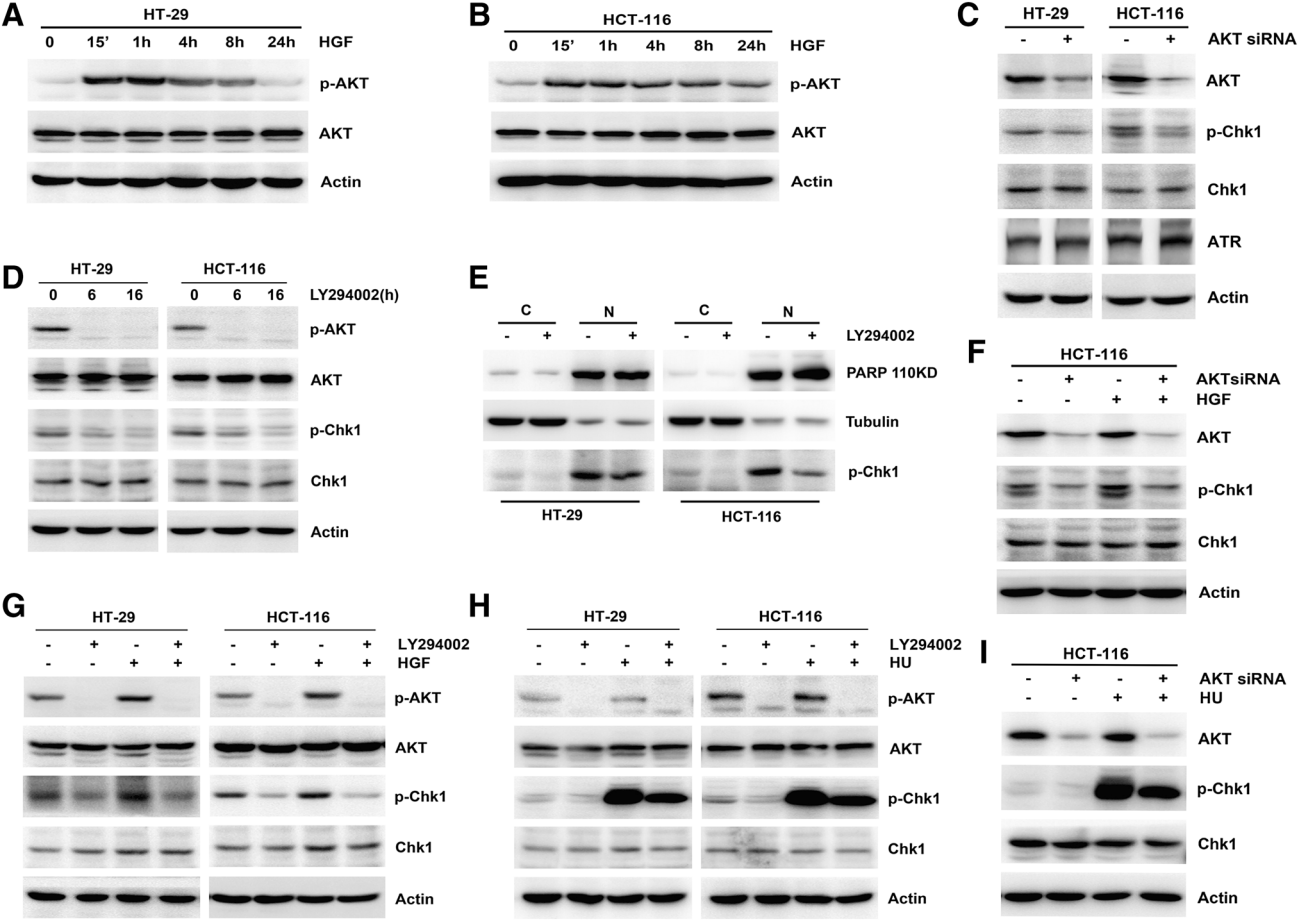
Effects of AKT on Chk1 phosphorylation. **a**, **b** HGF (25 ng/ml) was added to HT-29 and HCT-116 cells for the indicated times. The expression and phosphorylation of AKT was performed by Western blotting. **c** HT-29 and HCT-116 cells were transiently transfected with Scramble Control siRNA or AKT siRNA separately for 48 h. The expression of AKT, Chk1, ATR and Chk1 phosphorylation was performed by Western blotting. **d** HT-29 and HCT-116 cells were treated with 25 μM LY294002 for 6 and 16 h. The expression and phosphorylation of AKT and Chk1 was performed by Western blotting. **e** HT-29 and HCT-116 cells were treated with 25 μM LY294002 for 16 h, then subjected to subcellular fractionation. The expression of PARP, Tubulin and Chk1 phosphorylation in the cytosol (C) and nuclear (N) fractions was analyzed by Western blotting. **f** HCT-116 cells were transiently transfected with Scramble Control siRNA or AKT siRNA separately for 48 h, then added with 25 ng/ml HGF for 4 h. The expression of AKT, Chk1 and Chk1 phosphorylation of was performed by Western blotting. **g** HT-29 and HCT-116 cells were pretreated with 25 μM LY294002 for 16 h, then added with 25 ng/ml HGF for 4 h. The expression and phosphorylation of AKT and Chk1 was performed by Western blotting. **h** HT-29 and HCT-116 cells were pretreated with 25 μM LY294002 for 16 h, then added with 1 μM HU for 1 h. The expression and phosphorylation of AKT and Chk1 was performed by Western Blotting. **i** HCT-116 cells were transiently transfected with scramble control siRNA or AKT siRNA separately for 48 h, then added with 1 μM HU for 1 h. The expression of AKT, Chk1 and Chk1 phosphorylation was performed by Western blotting

To identify the key mediator of HGF-induced Chk1 phosphorylation, which also regulates TopBP1 and RAD51, we focused on AKT activity. As expected, HGF stimulated AKT activation, which was accompanied by Chk1 phosphorylation, at the indicated times in both HT-29 and HCT-116 cells (Fig. 4a, b). When AKT activity was suppressed by siRNA and an inhibitor, the basal level of p-Chk1 was gradually reduced (Fig. 4c, d). A subcellular fractionation experiment suggested that the AKT inhibitor attenuated Chk1 phosphorylation in both the nucleus and the cytoplasm (Fig. 4e). To further confirm the impact of AKT activity on Chk1 phosphorylation, HGF and HU were used to stimulate Chk1 activation after depletion of AKT activity. Decreasing AKT activity dramatically inhibited HGF- and HU-induced Chk1 activation (Fig. 4f–i). These results therefore confirmed that inhibiting AKT activity abolished Chk1 phosphorylation, including Chk1 auto-phosphorylation, and HU- and HGF-induced Chk1 activation.

### Depletion of AKT activity inhibited TopBP1 and RAD51 expression

**Fig. 5:**
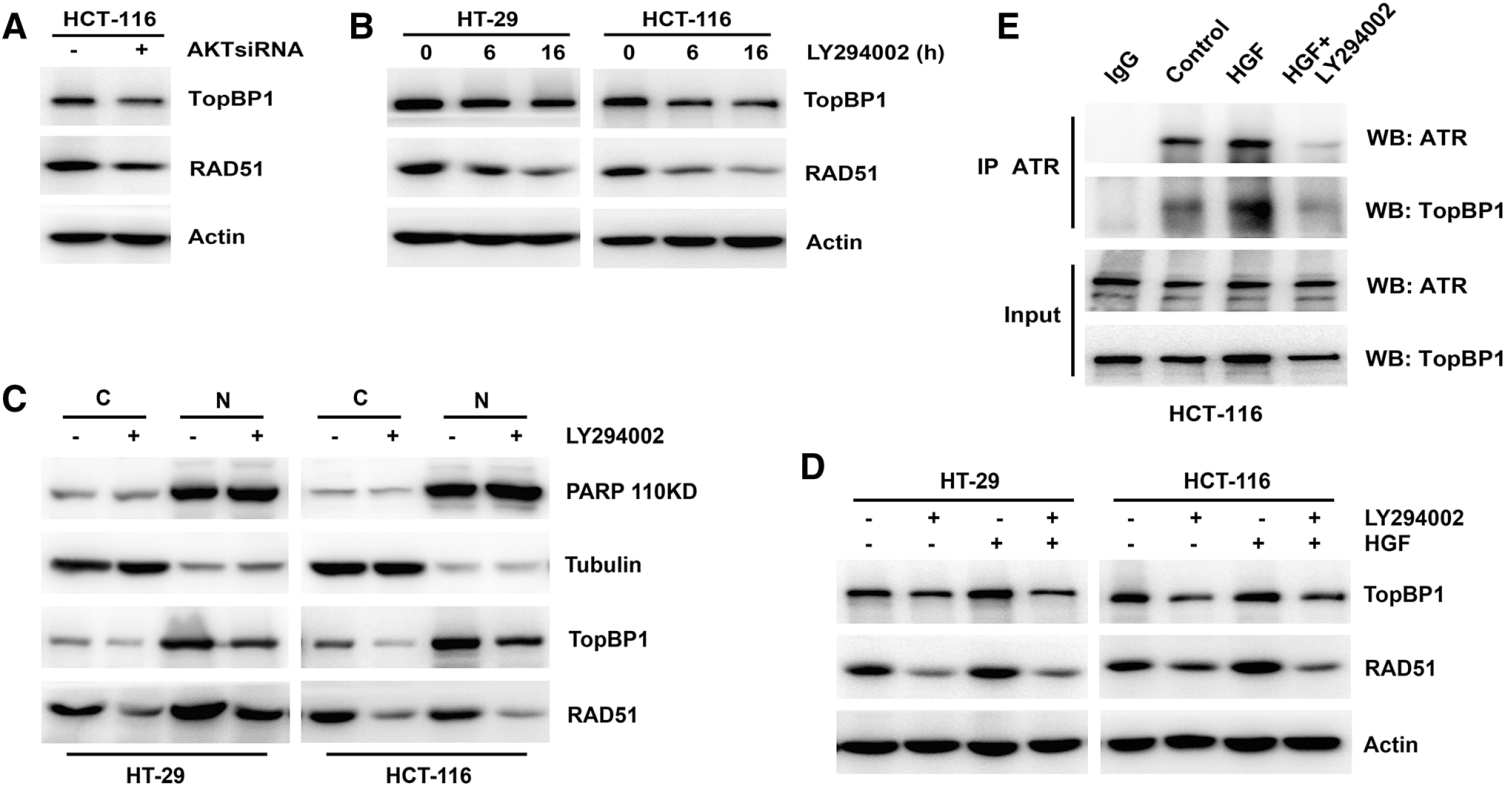
Effects of AKT on expression of TopBP1 and RAD51. **a** HCT-116 cells were transiently transfected with Scramble Control siRNA or AKT siRNA separately for 48 h. The expression of TopBP1 and RAD51 was performed by Western blotting. **b** HT-29 and HCT-116 cells were treated with 25 μM LY294002 for 6 and 16 h. The expression of TopBP1 and RAD51 was performed by Western blotting. **c** HT-29 and HCT-116 cells were treated with 25 μM LY294002 for 16 h, then subjected to subcellular fractionation. The expression of PARP, Tubulin, TopBP1, and RAD51 in the cytosol (C) and nuclear (N) fractions was analyzed by Western blotting. **d** HT-29 and HCT-116 cells were pretreated with 25 μM LY294002 for 16 h, then added with 25 ng/ml HGF for 4 h. The expression of TopBP1 and RAD51 was performed by Western blotting. **e** HCT-116 cells were pretreated with 25 μM LY294002 for 16 h, then added with 25 ng/ml HGF for 4 h. Whole-cell extracts from HCT-116 cells were immunoprecipitated with anti-ATR antibody. The immunoprecipitates were probed with ATR and TopBP1 antibodies. Control immunoprecipitation was done using IgG rabbit

Finally, we explored the association of AKT activity with TopBP1 and RAD51 expression in colon cancer cells. Inhibiting AKT activity with siRNA or an inhibitor suppressed total expression of TopBP1 and RAD51 (Fig. 5a, b). A subcellular fractionation assay indicated that levels of both cytoplasmic and nuclear TopBP1 and RAD51 were reduced in response to depleted AKT activity (Fig. 5c). To demonstrate the role of AKT activity in HGF-induced expression of TopBP1 and RAD51, cells were pretreated with an AKT inhibitor before being stimulated with HGF. Depletion of AKT activity attenuated the HGF-induced increase in TopBP1 and RAD51 expression (Fig. 5d). We also tested the effect of AKT activity on the HGF-induced enhancement of TopBP1–ATR complex formation. Immunoprecipitation indicated that decreasing AKT activity attenuated the enhancement of TopBP1–ATR complex formation (Fig. 5e). These results suggest that AKT activity was involved in the regulation of TopBP1 and RAD51, which directly interfered with Chk1 activity.

## Discussion

Chk1 is the major effector kinase activated by ATR. Increasing evidence suggests that Chk1 phosphorylation is implicated in cancer prognosis and sensitivity to anti-cancer therapy [2, 26]. Therefore, it is necessary to explore the modulation of Chk1 activity. Although emerging evidence has suggested that RTKs participate in the repair of damaged DNA and regulation of Chk1 phosphorylation, the function of growth factors in the regulation of Chk1 phosphorylation remains unknown. In this study, we demonstrated a novel role of HGF in the activation of Chk1 phosphorylation in colon cancer cells, regardless of RAS mutation status. Moreover, we showed that AKT activity mediated Chk1 activation via TopBP1 and RAD51 upregulation and TopBP1–ATR complex formation, which directly regulated Chk1 phosphorylation. These results provide solid evidence for an interaction between the HGF/MET and ATR/Chk1 pathways.

RTKs are frequently aberrant and upregulated in the majority of cancer types. Increasing evidence has demonstrated that the HER kinases MET and IGF-1R participate in DNA damage repair and G2 checkpoint regulation [12, 14, 16]. In the present study, we focused on the impact of the HGF/MET pathway on Chk1 activity, taking into account previous findings of a role of the MET pathway in regulation of cell proliferation and cetuximab resistance in colon cancer cells [15, 27]. Several studies have indicated that MET inhibition reduced DNA damage-induced activation of ATR and Chk1 in MET-overexpressing cells and p53-deficient cancer cells [17, 18]. However, whether HGF, the specific ligand of MET, is implicated in the regulation of Chk1 activity is not known. Therefore, we selected two colon cancer cell lines that are dependent on the ATR/Chk1 pathway, and observed that Chk1 phosphorylation levels gradually increased after HGF stimulation; this increase was not dependent on HGF concentration (Fig. 1a–c). Furthermore, we used siRNA to downregulate MET expression and thus confirmed that HGF-induced Chk1 activation occurred via MET activity (Fig. 1f). p-Chk1 localized to the nucleus and p-Chk1 in the cytoplasm exert different functions. Chk1 in the nucleus is activated by phosphorylation on Ser345 in response to DNA damage or when DNA replication is impeded. The activated Chk1 then dissociates from the chromatin into the nucleoplasm and degrades during checkpoint termination [2]. However, a proportion of the phosphorylated Chk1 can be exported to the cytoplasm after modification by other protein kinases. By performing subcellular fractionation, we further demonstrated that Chk1 phosphorylation mainly occurred in the nucleus in colon cancer cells, and HGF activated Chk1 in both the cytoplasm and the nucleus (Fig. 1d). Moreover, the elevated cytoplasmic p-Chk1 facilitated MET–Chk1 complex formation (Fig. 1e). The function of cytoplasmic Chk1 phosphorylation and MET–Chk1 complex formation deserves further study. Taken together, our findings provide sufficient evidence that the HGF/MET pathway positively regulated Chk1 activity. Although HGF and EGF are both important growth factors, they might play distinct roles in the regulation of DNA damage repair and Chk1 activity.

We speculated that Chk1 HGF-induced activation was directly promoted by essential adaptor proteins. First, we examined the expression of TopBP1 and Claspin, which contribute to Chk1 phosphorylation [28, 29]. The HGF-induced elevation in p-Chk1 levels was accompanied by an increase in TopBP1 expression, mainly in the nucleus, whereas Claspin expression levels remained stable (Fig. 2a–c). Downregulation of TopBP1 expression attenuated HU-induced p-Chk1 activation (Fig. 2e). Meanwhile, HGF promoted formation of the TopBP1–ATR replication complex, which led to ATR-dependent Chk1 phosphorylation (Fig. 2f). In addition to regulating a DNA damage checkpoint and a DNA replication checkpoint, Chk1 is also required for DNA repair during homologous recombination via interaction with the recombinase RAD51 [25]. HGF also increased RAD51 expression, which increased Chk1 activity (Fig. 3a–c), primarily in the nucleus (Fig. 3c).

To fully elucidate the link between HGF and Chk1 activity, we focused on AKT, which is the key protein regulating Chk1 nucleocytoplasmic transport and an essential downstream effector of the HGF/MET pathway [2, 15]. We used siRNA and an inhibitor to deplete AKT activity, and found that the basal level of p-Chk1 in both the nucleus and the cytoplasm was partially reduced in both HT-29 and HCT-116 cells (Fig. 4c–e). Furthermore, downregulating AKT activity likewise impaired HU- and HGF-induced phosphorylation-mediated activation of Chk1 (Fig. 4f–i), which indicates that AKT positively regulated Chk1 phosphorylation. Further investigation demonstrated that AKT depletion downregulated TopBP1 and RAD51 expression (Fig. 5a–c) and ATR–TopBP1 complex formation (Fig. 5d), all of which directly regulated Chk1 activity. When considering the regulation of Chk1 by AKT, most studies have suggested that AKT inhibition restored Chk1 activation [19, 21]. However, under exposure to DNA-damaging agents, the activity of both Chk1 and AKT was enhanced [30, 31]. Moreover, immunohistochemistry revealed that cytoplasmic expression of p-Chk1 was positively associated with AKT expression in breast cancer tissues [6]. AKT is a vital regulator of various pathways and exhibits complex regulatory functions; therefore, its role in regulating Chk1 activity might be complicated and diverse. Here, we have demonstrated that AKT played a critical role in HGF-induced Chk1 activation; determining whether other factors take part in this process requires further study. The present study may shed new light on the molecular mechanisms underlying regulation of Chk1 activity by AKT.

## Conclusion

In summary, our study demonstrated that HGF induced Chk1 phosphorylation. This was accompanied by increased TopBP1 and RAD51 expression and enhanced TopBP1–ATR complex formation, which mediated the regulation of Chk1 phosphorylation. In this process, AKT activity directly enhanced HGF-induced TopBP1 and RAD51 expression, and ATR–TopBP1 complex formation. These results demonstrate the underlying molecular mechanisms by which HGF regulates Chk1 activity, which might have clinical significance for prognosis and drug sensitivity in cancer therapy.

## Abbreviations

ATM: Ataxia-telangiectasia mutated
ATR: ATM and Rad3-related
Chk2: Checkpoint kinase 2
Chk1: Checkpoint kinase 1
DDR: DNA damage repair
HR: Homologous recombination
RTKs: Receptor tyrosine kinases
HGF: Hepatocyte growth factor
RS: Replication stress
DSBs: Double-strand breaks
ssDNA: Single-stranded DNA
HU: Hydroxyurea
RPMI: Roswell Park Memorial Institute
